# The TIR/BB-loop mimetic AS-1 prevents Ang II-induced hypertensive cardiac hypertrophy via NF-κB dependent downregulation of miRNA-143

**DOI:** 10.1038/s41598-019-42936-x

**Published:** 2019-04-23

**Authors:** Juan Song, Qifei Xie, Lin Wang, Yi Lu, Peijing Liu, Ping Yang, Rui Chen, Chen Shao, Chen Qiao, Zhongqun Wang, Jinchuan Yan

**Affiliations:** 1 Department of Cardiology, Affiliated Hospital of Jiangsu University, Zhenjiang, Jiangsu Province 212001 P.R. China; 2 Department of Cardiology, The Fourth Affiliated Hospital of Jiangsu University, Zhenjiang, Jiangsu Province 212001 P.R. China; 3 Department of Clinical Pharmacy, Affiliated Hospital of Jiangsu University, Zhenjiang, Jiangsu Province 212001 P.R. China

**Keywords:** Cardiology, Cardiac hypertrophy

## Abstract

Untreated pathological cardiac hypertrophy, which can be caused by sustained systemic hypertension, may lead to heart failure. In the present study, we investigated whether AS-1 had attenuating effects on hypertension-induced cardiac hypertrophy, and whether this process was mediated by the regulation of miRNA-143. To induce the hypertrophic response *in vitro*, cardiomyocytes were stimulated with Ang II for 24hs. AS-1 administration strongly attenuated Ang II-induced hypertrophic response of cardiomyocytes. Chronical infusion of Ang II via implanted osmotic mini-pump induced increased blood pressure and cardiac hypertrophy *in vivo*. AS-1 administration attenuated hypertension-induced cardiac hypertrophy by, at least in part, inhibin of MAPK signaling. We observed, for the first time, upregulated expression of miRNA-143 in Ang II-induced cardiomyocytes, and inhibition of miRNA-143 significantly reduced the Ang II-induced hypertrophic responses. Importantly, AS-1 administration diminished the Ang II-induced upregulation of miRNA-143. Overexpression of miRNA-143 abolished the attenuating effects of AS-1 on Ang II-induced hypertrophic response of cardiomyocytes. Additionally, AS-1 administration abrogates Ang II-induced nuclear translocation of p50 NF-κB subunit in hypertrophic cardiomyocytes. Application of NF-κB inhibitor significantly suppressed Ang II-induced upregulation of miRNA-143. Our data suggest a novel mechanism by which AS-1 attenuates Ang II-induced hypertrophic response through downregulation miRNA-143 expression in a NF-κB-dependent manner.

## Introduction

Cardiac hypertrophy is an adaptive response of many cardiac diseases, including hypertension, valvular dysfunction and myocardial infarction^1,2^. Among these diseases, hypertrophic responses are initially induced by pressure or volume overload^3^. In respond to the pressure overload, the terminally-differentiated cardiomyocytes undergo abnormal enlargement and thickening, which is accompanied by reprogramming of fetal genes, including natriuretic peptides (NPs) and β-myosin heavy chain (β-MHC)^4^. In almost all cases, the sustained and uncontrolled cardiac hypertrophy will eventually result in contractile dysfunction and heart failure. Unfortunately, there are no effective therapeutic interventions available for hypertrophic cardiomyopathy at present.

MicroRNAs (miRNAs) are a class of 18–24 nucleotide, noncoding RNAs that inhibit the expression of specific mRNA targets through base-paring^5^. Expressed ubiquitously or in a tissue-specific manner, miRNAs regulate a series of biological and pathological events, including organ development, cells proliferation/death and cell differentiation^6,7^. During the development of mammalian hearts, biosynthesis of miRNAs, including miRNA-143/145, is essential by promoting cell differentiation^8^. In addition to their functions in cardiovascular development, a wealth of recent studies demonstrated dysregulated expression of miRNAs in many cardiovascular diseases^9,10^. Particularly, Ward A. Heggermont and colleagues showed that inhibition of miRNA-146a provides protection against the pressure overload-induced cardiac hypertrophy and dysfunction^11^. Additionally, constitutive null mutation of miRNA-133 successfully induced cardiac hypertrophy in mouse^12^. There does seem to be increasing evidence that the abnormally regulated expression of miRNAs contributes, at least in part, to the development and progression of cardiac hypertrophy.

Hypertension has been known as an important determinant of left ventricular hypertrophy^13^. Although the mechanisms of hypertension to cardiac hypertrophy are still elusive, overwhelming evidence indicates that altered expressions of miRNAs might be involved in increased blood pressure^5^. miRNA-143 has been identified as an essential player in hypertension by regulating vascular smooth muscle cells (VSMCs) activities. Knockout of miRNA-143 and 145 resulted in reduced blood pressure in mouse due to impaired vascular tone^14^. In parallel, Thomas Boettger and colleagues observed that deficiency of miRNA-143 incapacitated contractile abilities of VSMCs^15^. Recently, miRNA-143 was found to regulate the crosstalk between smooth muscle cells and endothelial cells and play a critical role in pulmonary arterial hypertension^16^. However, no report has investigated the function of miRNA-143 in the pathological process of hypertension-induced cardiac hypertrophy.

Several studies suggested that hydrocinnamoyl-L-valyl pyrrolidine (AS-1), a synthetic mimetic of TIR/BB-loop, has protective effects against cardiovascular diseases^17–19^. Our previous studies reported that the application of AS-1 prevented the transverse aortic constriction (TAC)-induced hypertrophy *in vivo* through blocking the activity of transcription factor NF-κΒ and MAPK activation^18^. However, current studies of therapeutic effects of AS-1 on cardiac hypertrophy are exclusively using the TAC-induced hypertrophic mouse model without affecting blood pressure^18^. In the present study, we utilized a chronic hypertensive cardiac hypertrophy mouse model by infusion of Angiotensin II (Ang II). Administration of Ang II, which has direct effects on myocardium and induces hypertension, is an established method to induce hypertensive cardiac hypertrophy^20,21^. We examined the effects of AS-1 on hypertension and cardiac hypertrophy response *in vivo*, which is barely described in previous studies. We also investigated the expression of miRNA-143 in Ang II-induced hypertensive cardiac hypertrophy, and further revealed that miRNA-143 is an essential mediator in hypertensive cardiac hypertrophy. Importantly, AS-1 administration attenuated Ang II-induced hypertrophy by suppressing NF-κB-dependent miRNA-143 expression.

## Results

### AS-1 reduces Ang II-induced hypertension

**Figure 1:**
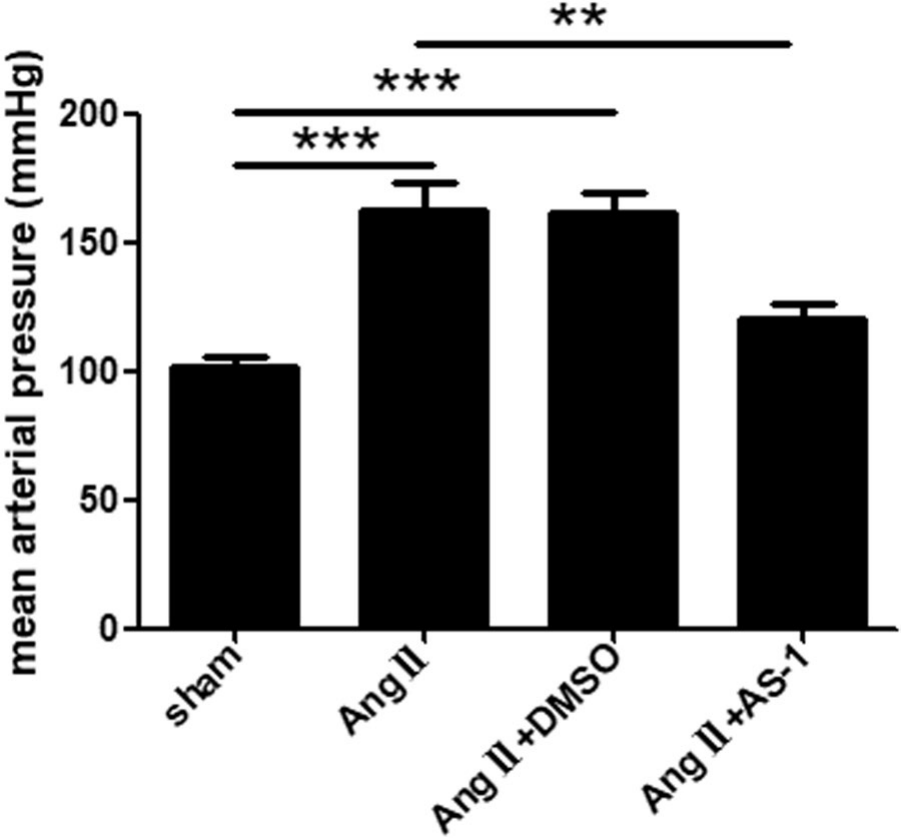
AS-1 reduces Ang II-induced hypertension *in vivo*. Mean arterial pressure among each group was assessed by tail cuff blood pressure system. sham: mice treated with saline. Ang II: mice treated with Ang II. Ang II + DMSO: mice induced by Ang II and i.p. injected with DMSO. Ang II + AS-1: mice induced by Ang II and i.p. injected with AS-1. Mean ± SEM, n = 6, **p < 0.01, ***p < 0.001.

After infusion of Ang II for 4 weeks, we observed significantly increased blood pressures *in vivo*, as compared with age-matched sham control mice (Fig. 1). As shown in Fig. 1, administration of AS-1 significantly reduced tail blood pressure by 26.17% in Ang II-induced hypertensive mice, while administration of DMSO showed no difference in tail blood pressure, indicating AS-1 reduced Ang II-induced hypertension *in vivo*.

### AS-1 reverses Ang II-induced cardiac hypertrophy

**Figure 2:**
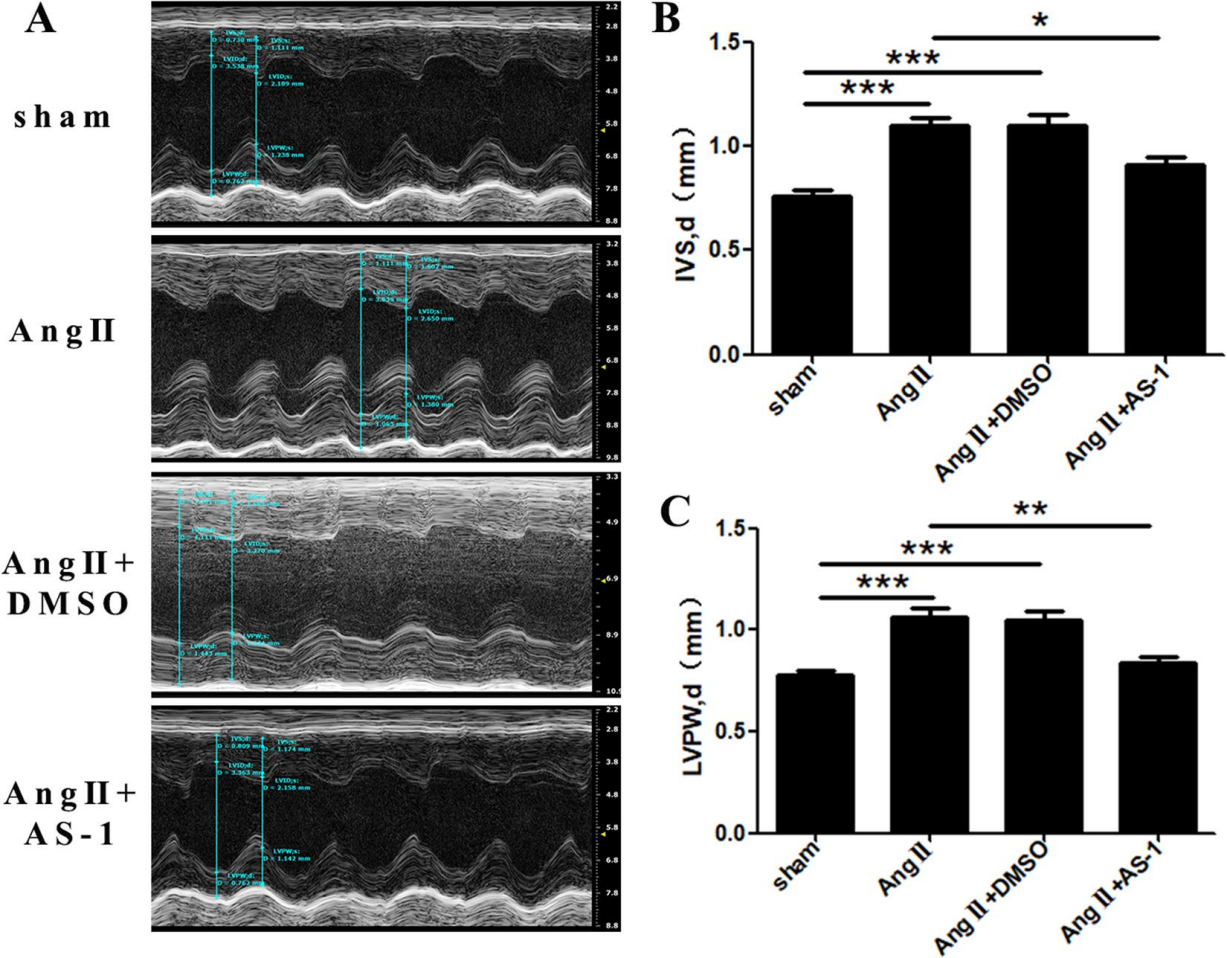
AS-1 attenuates Ang II-induced increases in left ventricular wall thickness *in vivo*. Mice were continuously administrated with either Ang II or normal saline using a mini-pump for 28 days. 3 days before which, mice were intraperitoneally injected with AS-1. Interventricular septum diastolic dimension (IVS, d) and left ventricular posterior wall diastolic dimension (LVPW, d) were examined by two-dimensional echocardiography. (**A**) Representative echocardiographic M-mode images from six consecutive cardiac cycles. IVS, d (**B**) and LVPW, d (**C**) were measured and compared among each group. sham: mice treated with saline. Ang II: mice treated with Ang II. Ang II + DMSO: mice induced by Ang II and i.p. injected with DMSO. Ang II + AS-1: mice induced by Ang II and i.p. injected with AS-1. Mean ± SEM, n = 6, *p < 0.05, **p < 0.01, ***p < 0.001.

To further address whether AS-1 could attenuate hypertension-induced cardiac hypertrophy *in vivo*, we measured the thickness of ventricular wall by two-dimensional echocardiography. As shown in Fig. 2A, interventricular septum diastolic dimension (IVS, d) and left ventricular posterior wall diastolic dimension (LVPW, d) of Ang II-induced mice were increased by 44.04% (0.764 ± 0.026 vs 1.100 ± 0.041) (Fig. 2B) and 37.60% (0.774 ± 0.024 vs 1.065 ± 0.042) (Fig. 2C) respectively, when compared with age-matched sham control mice, indicating cardiac hypertrophy was induced by Ang II infusion. However, the IVS, d and LVPW, d of Ang II-induced hypertrophic mice were significantly decreased by 17.07% (Fig. 2B) and 21.06% (Fig. 2C) respectively upon the administration of AS-1.

**Figure 3:**
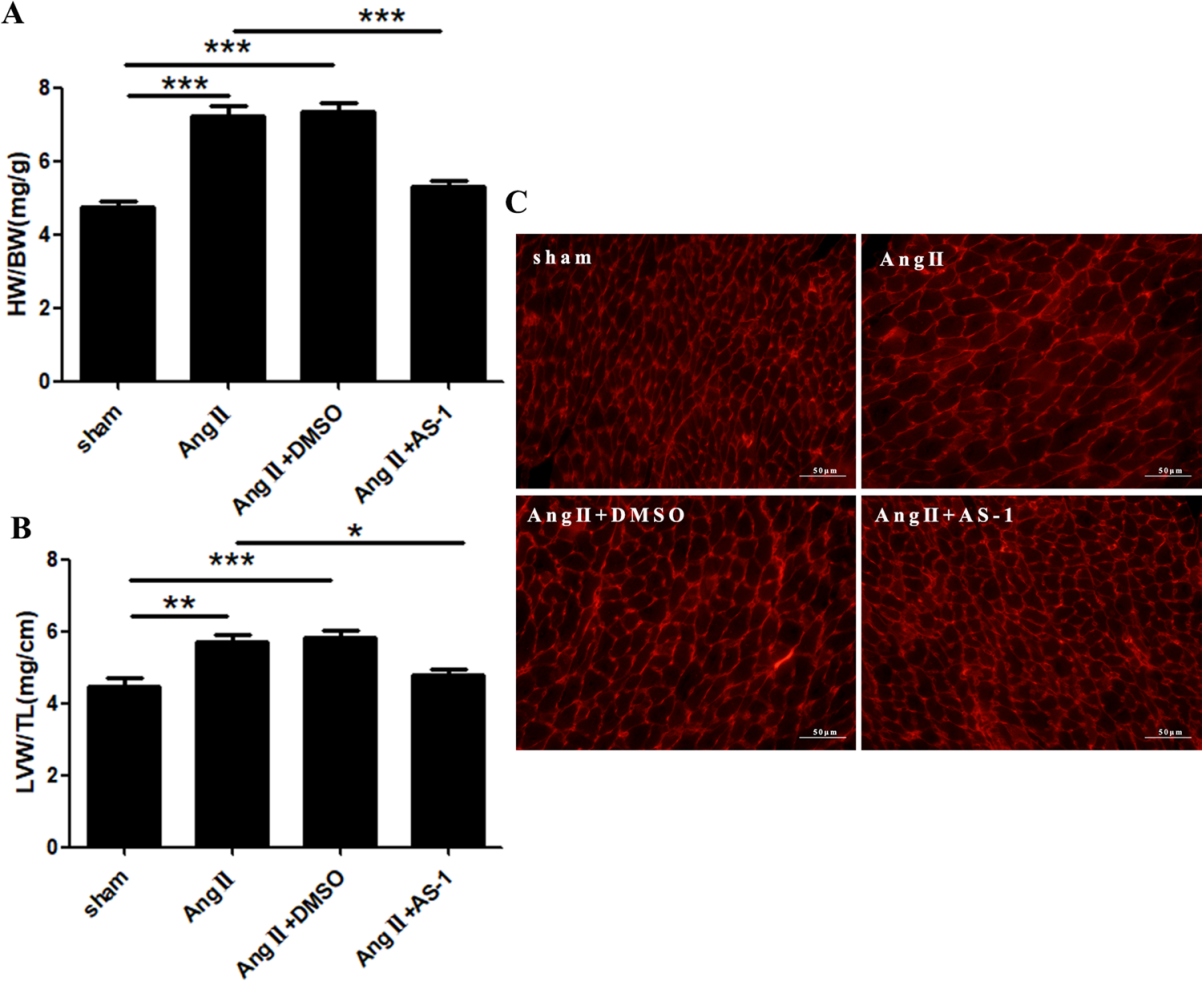
AS-1 attenuates Ang II-induced cardiac hypertrophy *in vivo*. Mice were continuously administrated with either Ang II or normal saline using a mini-pump for 28 days. 3 days before which, mice were intraperitoneally injected with AS-1. Hearts were harvested and weighted. (**A**) The ratio of HW/BW among each group was calculated and compared. (**B**) The ratio of LVW/TL among each group was calculated and compared. (**C**) Representative images of WGA staining on paraffin-embedded heart sections from each group. sham: mice treated with saline. Ang II: mice treated with Ang II. Ang II + DMSO: mice induced by Ang II and i.p. injected with DMSO. Ang II + AS-1: mice induced by Ang II and i.p. injected with AS-1. Mean ± SEM, n = 6, *p < 0.05, **p < 0.01, ***p < 0.001.

Additionally, our data showed that the ratio of HW/BW and LVW/TL of Ang II-induced mice were dramatically upregulated by 53.01% (4.733 ± 0.161 vs 7.242 ± 0.274) (Fig. 3A) and 27.70% (4.477 ± 0.226 vs 5.717 ± 0.206) (Fig. 3B), respectively, as compared with age-matched sham control mice. In contrast, AS-1 treatment obviously reversed the hypertrophic response, indicated by downregulating the ratio of HW/BW (Fig. 3A) and LVW/TL (Fig. 3B) by 26.77% and 16.60% as compared with that of Ang II-induced mice. Consistently, wheat germ agglutinin (WGA) staining showed that AS-1 administration significantly reversed the enlargement of cell size in the heart of Ang II-induced mice (Fig. 3C). Collectively, the data suggest AS-1 could attenuate, at least in part, the hypertension-induced cardiac hypertrophy *in vivo*.

### AS-1 attenuates Ang II-induced hypertrophic response of cardiomyocytes by inhibition of MAPK signaling

**Figure 4:**
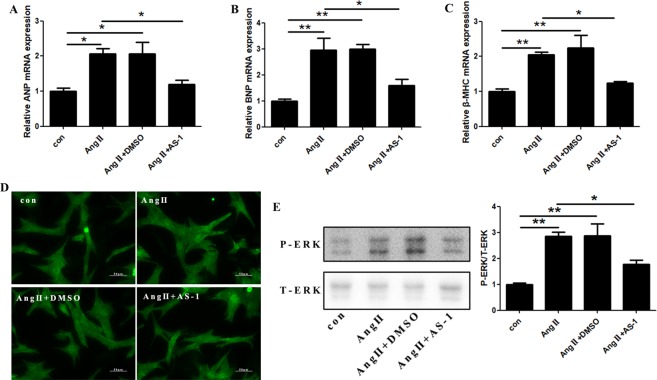
AS-1 attenuates Ang II-induced hypertrophic response of cardiomyocytes by inhibition of ERK1/2 phosphorylation *in vitro*. Rat primary cardiomyocytes were stimulated with Ang II (0.1 μM) for 24 hours. AS-1 (100 μM) or DMSO was administrated 30 minutes before Ang II induction. (**A**–**C**) The mRNA expression of ANP (**A**), BNP (**B**) and β-MHC (**C**) in primary cardiomyocytes was measured in each group. (**D**) Representative images of primary cardiomyocytes stained with anti-actinin, a marker for α-cardiac muscle actinin, by immunofluorescence. (**E**) Representative images of total ERK1/2 and phosphor-ERK1/2 protein levels in primary cardiomyocytes by western blot. con: non-treated cells. Ang II: cells induced by Ang II. Ang II + DMSO: cells administrated with DMSO followed by Ang II induction. Ang II + AS-1: cells administrated with AS-1 followed by Ang II induction. Mean ± SEM, n = 4, *p < 0.05, **p < 0.01.

To further confirm the protection of AS-1 on Ang II-induced cardiac hypertrophy, rat primary cardiomyocytes were subjected to Ang II (0.1 μM) for 24 hours. As shown in Fig. 4A–C, the mRNA level of ANP, BNP and β-MHC was elevated by 106.3% (1.000 ± 0.085 vs 2.063 ± 0.154), 195% (1.000 ± 0.083 vs 2.950 ± 0.47) and 104.3% (1.000 ± 0.073 vs 2.043 ± 0.079) respectively in Ang II-induced rat primary cardiomyocytes as compared with untreated control cells. In addition, hypertrophic response of cardiomyocytes was examined by cell size. We observed that the cell size of Ang II-induced rat primary cardiomyocytes was obviously increased when compared with untreated control cells, indicating a hypertrophic response of rat primary cardiomyocytes was induced by Ang II administration, while AS-1 pre-treatment obviously decreased the size of cultured cardiomyocytes (Fig. 4D). It has been reported that Ang II-induced hypertrophy of cardiomyocytes is mediated by MAPK pathway activation^22^. Next, we evaluated the phosphorylation of ERK1/2 after AS-1 administration by western blot. As shown in Fig. 4E, we observed increased phosphorylation of ERK1/2 in Ang II-induced hypertrophic cardiomyocytes. Interestingly, AS-1 significantly suppressed the phosphorylation of ERK1/2.

### miRNA-143 is required for Ang II-induced hypertrophy of cardiomyocytes

**Figure 5:**
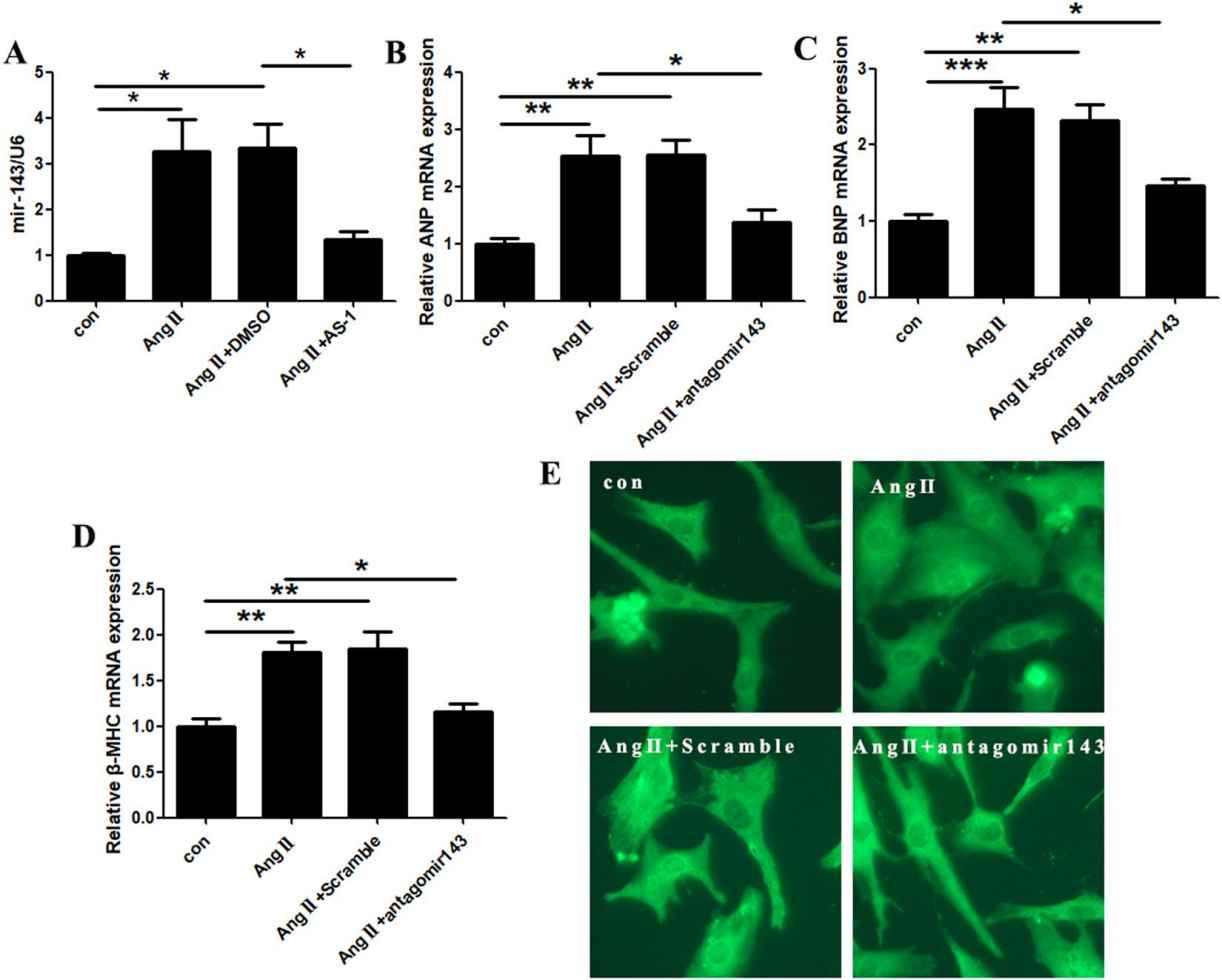
AS-1 suppresses Ang II-induced increases of miRNA-143 in cardiomyocytes *in vitro*. MiRNA-143 expressions were evaluated in Ang II-induced primary cardiomyocytes with or without AS-1 administration. MiRNA-143 expression was inhibited by transfection of its specific antagomir143 or scramble control. (**A**) The level of miRNA-143 expression was evaluated and compared among each group. mRNA levels of ANP (**B**), BNP (**C**) and β-MHC (**D**) in primary cardiomyocytes examined among each group. (**E**) Representative images of primary cardiomyocytes stained with anti-actinin, a marker for α-cardiac muscle actinin, by immunofluorescence. con: non-treated cells. Ang II: cells induced by Ang II. Ang II + DMSO: cells administrated with DMSO and induced by Ang II. Ang II + AS-1: cells administrated with AS-1 and induced by Ang II. Ang II + Scramble: cells transfected with scramble siRNA and induced by Ang II. Ang II + antagomir143: cells transfected with antagomir143 and induced by Ang II. Mean ± SEM, n = 4, *p < 0.05.

Previous studies have showed miRNA-143 participates the development of mammalian hearts and is required to maintain the vascular hemostasis^5,6^. miRNA-143 has also been demonstrated existing in the heart^23^. Interestingly, we found a significantly enhanced expression of miRNA-143 by 227.5% in primary rat cardiomyocytes after Ang II induction for 24 hours (Fig. 5A).

To further investigate the involvement of miRNA-143 in Ang II-induced hypertrophic responses, we inhibited miRNA-143 by transfection with its specific antagomir (antagomir-143) in rat primary cardiomyocytes. As shown in Fig. 5B–D, inhibition of miRNA-143 significantly downregulated Ang II-induced elevations of ANP, BNP and β-MHC mRNA expressions by 45.94%, 40.76%, 36.84%, respectively. Consistently, antogomir-143 obviously rescued the increased size of Ang II-treated cardiomyocytes (Fig. 5E). The data indicate that upregulated expression of miRNA-143 is required in Ang II-induced hypertrophic responses of cardiomyocytes.

### AS-1 attenuates Ang II-induced hypertrophy by suppressing miRNA-143 expression

**Figure 6:**
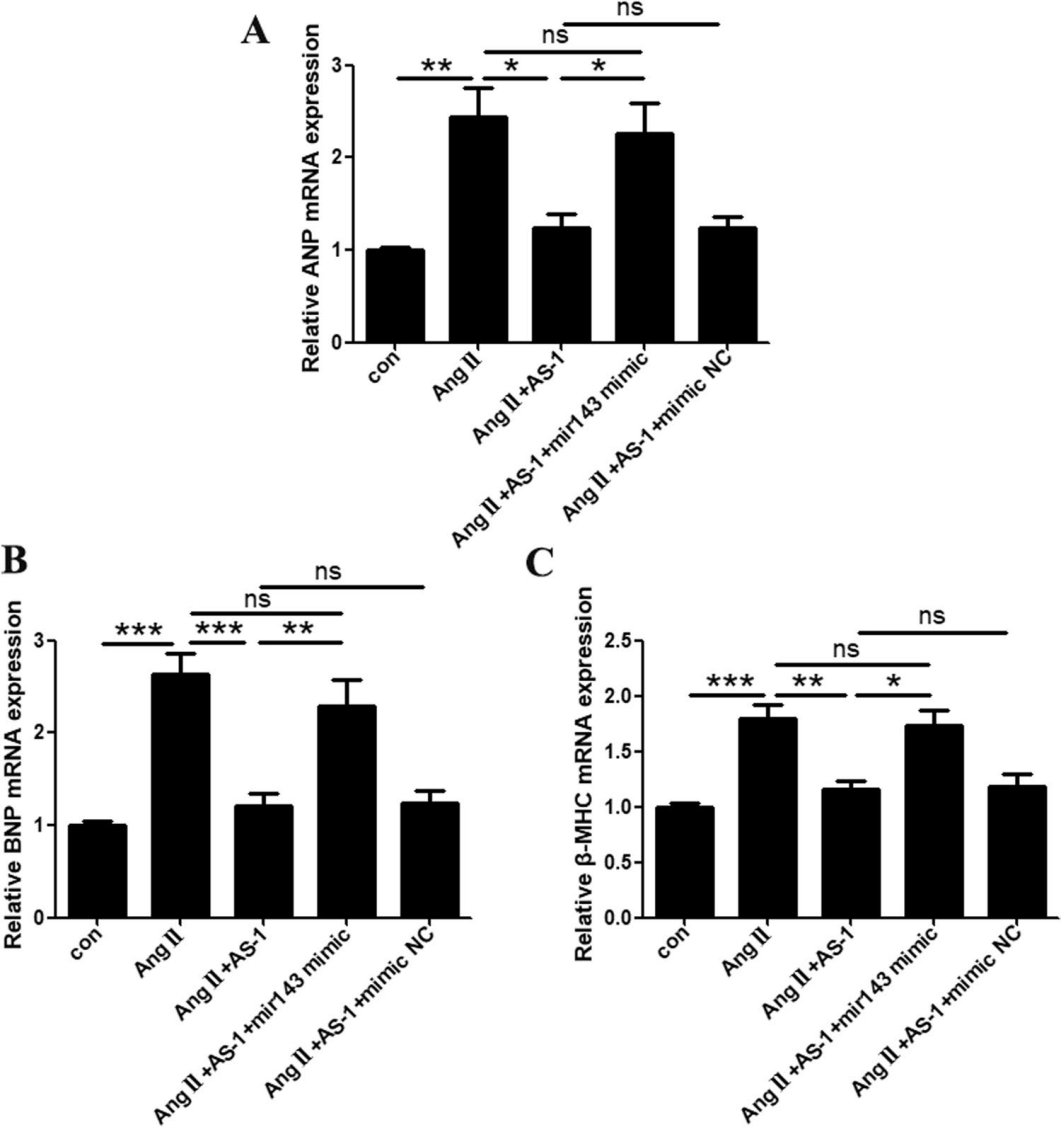
Overexpression of miRNA-143 abrogates AS-1-attenuated hypertrophic responses in primary cardiomyocytes. miRNA-143 was overexpressed in primary cardiomyocytes via transfection. Cells transfected with non-specific control miRNA (scramble) was used as control. The mRNA expression level of ANP (**A**), BNP (**B**) and β-MHC (**C**) was examined via qRT-PCR and compared among each group. con: non-treated cells. Ang II: cells induced by Ang II. Ang II + AS-1: cells administrated with AS-1 followed by Ang II induction. Ang II + AS-1 + miRA-143 mimic: miRNA-143-overexpressed cells administrated with AS-1 and induced by Ang II. Ang II + AS-1 + mimic NC: non-specific control miRNA transfected cells administrated with AS-1 and induced by Ang II. Mean ± SEM, n = 4, *p < 0.05, **p < 0.01, ***p < 0.001. ns: no significance.

Firstly, we examined whether AS-1-attenuated cardiac hypertrophy was mediated by reducing miRNA-143 expression. As shown in Fig. 5A, AS-1 treatment significantly suppressed Ang II-stimulated elevation of miRNA-143 expression by 59.30%, as compared with Ang II-stimulated cardiomyocytes, while no changes were detected in vehicle control (DMSO)-treated cells. Next, we overexpressed miRNA-143 in primary cardiomyocytes by transfection of synthesized miRNA-143. As shown in Fig. 6A–C, mRNA expressions of ANP, BNP and β-MHC were significantly upregulated in Ang II-induced cardiomyocytes as compared with sham control cells (144.3%, 163.8% and 79.5% respectively). The administration of AS-1 greatly downregulated the expression of ANP, BNP and β-MHC by 49.12%, 54.21% and 35.21% respectively. However, the over-expression of miRNA-143 abolished the effects of AS-1 on attenuating Ang II-induced upregulation of ANP, BNP and β-MHC expression, while the transfection of non-specific control miRNA showed no significant differences. The data indicate that one of the possible mechanisms by which AS-1 attenuated Ang II-induced cardiac hypertrophy is suppressing the expression of miRNA-143.

### AS-1 suppresses miRNA-143 expression by inhibition of NF-κB

**Figure 7:**
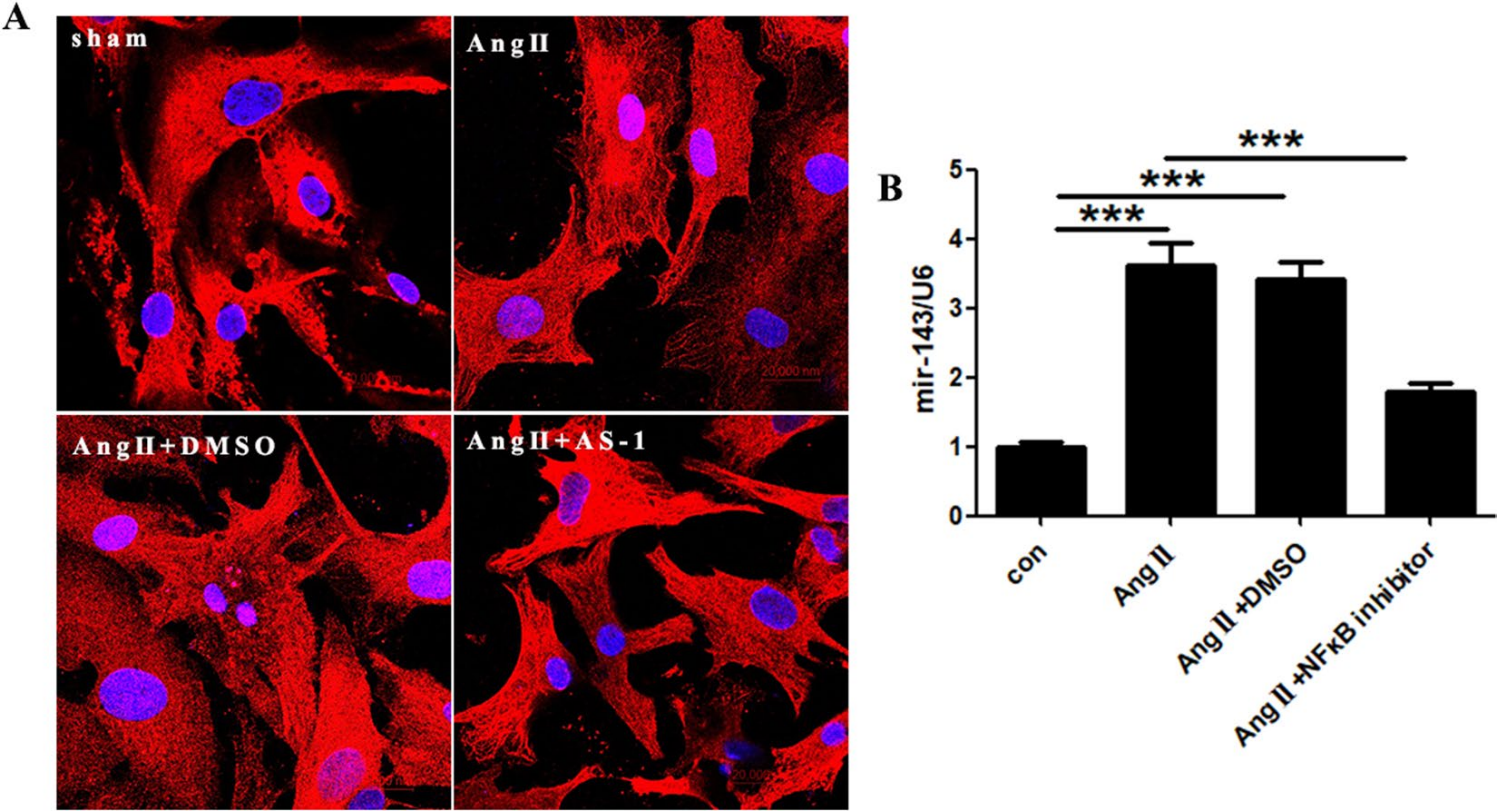
AS-1 reduces miRNA-143 expression by inhibiting p50 NF-κB nuclear localization. (**A**) Immunostaining of p50 NF-κB subunit in primary cardiomyocytes and images were captured using confocal microscope. (**B**) miRNA expressions was examined in cardiomyocytes and compared among each group. con: non-treated cells. Ang II: cells induced by Ang II. Ang II + DMSO: cells administrated with DMSO followed by Ang II induction. Ang II + AS-1: cells administrated with AS-1 followed by Ang II induction. Ang II + NF-κB inhibitor (BAY 11-7082): cells administrated with NF-κB inhibitor (BAY 11-7082) for 12 hours followed by Ang II induction. Mean ± SEM, n = 4, *p < 0.05, **p < 0.01, ***p < 0.001. ns: no significance.

Previous studies indicate the NF-κB, a transcription factor, is tightly involved in the regulation of miRNA expression, leading us to investigate whther NF-κB is a putative mediator in the AS-1 regulated miRNA-143 expression in Ang II-stimulated cardiomyocytes. Firstly, we examined the localization of p50, a subunit of NF-κB, with or without AS-1 treatment in Ang II-stimulated cardiomyocytes. We observed promoted nuclear translocation of p50 in cardiomyocytes upon Ang II induction as compared with sham control cardiomyocytes (Fig. 7A). However, the nuclear translocation of p50 was abrogated when cardiomyocytes were subjected to AS-1 administration *in vitro*, suggesting the involvement of NF-κB in AS-1-attenuated Ang II-induced cardiac hypertrophic response. Next, we investigated whether Ang II-induced increase of miRNA-143 can be diminished by inhibition of NF-κB. As shown in Fig. 7B, NF-κB inhibitor (BAY 11-7082) significantly suppressed Ang II-induced upregulation of miRNA-143. Taken together, the data demonstrate that AS-1 inhibited the activation of NF-κB, which abrogated NF-κB-dependent upregulation of miRNA-143 upon Ang II stimulation in cardiomyocytes.

## Discussion

Pathological cardiac hypertrophy, a maladaptive response to pressure/volume overload, is characterized by thickening of the myocardium^24^. Hypertension is one of the most common causes of hypertrophic cardiomyopathy^25^. It is well-accepted that management of hypertensive cardiac diseases can not only prevent left ventricular hypertrophy but slow down the development of heart failure^26^. To understand the pathological process of hypertension to cardiac hypertrophy, studies have been focused on signaling pathways that control cell size expansion and apoptosis, including MAPK^27^. Except for classical proteins and signaling pathways, more studies suggest that the epigenetic modifications, including DNA modification and miRNAs biosynthesis, may contribute to the hypertension-induced cardiac hypertrophy^28^. In recent years, there has been overwhelming evidence that miRNA-143 are essential regulators in various cardiovascular diseases, due to their functions in modulating the phenotypes of vascular smooth muscle cells (VSMCs)^5,14,29^. However, there is barely evidence shows the involvement of miRNA-143 in hypertensive cardiac hypertrophy. In the current study, we utilized an Ang II-induced hypertrophy model. Ang II plays a critical role in the development and maintenance of cardiac hypertrophy^30^. The underlying machinery of Ang II-induced hypertrophic cardiomyopathy is partly mediated by its hemodynamic effects. The hypertrophic response was observed in Ang II-induced hypertensive mice in an *in vivo* study conducted by Dikalova A and colleagues^31^. Consistently, we observed increased blood pressure and left ventricular hypertrophic phenotypes after Ang II administration.

A significant result of our study was that miRNA-143 was upregulated in Ang II-induced hypertrophy. Dysregulation of miRNA-143 has been related to various cardiac diseases^5^. In a comprehensive examination of miRNA expression in human congestive heart failure, Scot J. Matkovich and colleagues found upregulated expressions of twenty-eight miRNAs by at least 2-fold change. Specifically, miRNA-143 was significantly upregulated by 9-fold change in heart failure specimens as compared with non-failing hearts, suggesting miRNA-143 can be used as an indicator of end-stage heart failure^32^. We found miRNA-143 expression was dramatically upregulated after the induction of hypertension-induced cardiac hypertrophy. Importantly, inhibition of miRNA-143 expression significantly reduced hypertrophic responses of cardiomyocytes. This discovery revealed a novel function of miRNA-143 in hypertension-induced hypertrophy, which might be used as valuable biomarker for cardiac hypertrophy.

AS-1, a synthetic low-molecule mimetic of TIR/BB-loop, was first synthesized by Tamas Bartfai and colleagues^33^. Since then, the function of AS-1 has been investigated in many pathological conditions, including IL-1β-induced fever and steatohepatitis^33,34^. The presence of AS-1 competes with MyD88, resembling the effect of null mutation of MyD88 on subsequent pathways. MyD88 has been revealed as an adapter protein in Toll-like receptor (TLR)-and interleukin-1 receptor (IL-1R)-induced activation of NF-κB, which leads us to evaluate the expression of NF-κB activity in hypertrophic cardiomyocytes upon AS-1 administration^35^. Previously, we demonstrated a function of AS-1 in attenuating TAC-induced cardiac hypertrophy through inhibiting IL-1R-mediated MyD88-dependent signaling^18^. In the present study, we further investigated the function of AS-1 in attenuating Ang II-induced hypertrophic responses. Our data demonstrate that AS-1 is sufficient to ameliorate Ang II-induced hypertrophy of cardiomyocytes though inhibiting abnormally-upregulated miRNA-143 expression in a NF-κB-dependent manner. In addition, we found that AS-1 could improve Ang II-induced hypertension *in vivo*. Although the underlying mechanism by which AS-1 decreased blood pressures in Ang II-treated mice was not explored, our data provide an idea that AS-1 might have powerful potentials in the treatment of cardiac hypertrophy induced by hypertension.

There are several limitations to our study. We demonstrated that miRNA-143 is significantly upregulated in hypertension-induced cardiac hypertrophy; however, the specific mechanism of how dysregulated miRNA-143 expression contributes to the hypertrophic responses needs further investigation. Although we found that AS-1 reduced Ang II-induced blood pressure increases, whether AS-1 functions by manipulating the expression of miRNA-143 remains unknown and could be an interesting topic for future study.

In summary, our findings demonstrated that administration of AS-1 attenuated Ang II-induced cardiac hypertrophy both *in vitro* and *in vivo*. *In vitro* studies suggested a novel mechanism that AS-1 attenuates hypertension-induced cardiac hypertrophy is mediated by downregulation of miRNA-143 in a NF-κB-dependent manner, which could be a new therapeutic target in the treatments of hypertension-induced cardiac hypertrophy.

## Materials and Methods

### Synthesis of TIR/BB-loop mimetics AS-1

AS-1 was synthesized as described previously^18^. AS-1 was obtained as pale yellowish oil and dried in vacuo for 24 hours. The structure of AS-1 was examined by nuclear magnetic resonance (1 H NMR): (500 MHz, MeOD): 7.34–7.17(m, 5 H), 6.24 (d, J = 8.4 Hz, 1 H), 4.60, 4.58 (dd, J = 6.8 Hz, 7.2 Hz, 1 H) 3.74–3.70(m, 1 H), 3.49–3.40(m, 3 H), 2.99–2.92(m, 3 H), 2.53(t, J = 8.0 Hz, 1 H), 1.97–1.83(m, 5 H), 0.90 (d, J = 6.8 Hz, 3 H), 0.82 (d, J = 6.8 Hz, 3 H); LRMS (EI, 70 eV) m/z(%): 302 (M + 5), 260(2), 204(14), 154(5), 127(4), 105(8), 91 (14), 72(100). The crystals of AS-1 were dissolved and prepared in DMSO. No significant-cytotoxicity of AS-1 was observed by cell viability (MTT) assay (data not shown).

### Experimental mice

Male C57BL/6 mice, 6–8 weeks, weighted 18–22 g, were provided by the Laboratory Animal Research Center of Jiangsu University (Zhenjiang, China). 3–5 mice/cage were bred and kept under defined-flora, pathogen-free conditions in the animal research center under an artificial 12/12 light-dark cycle and with room temperature of 18 °C to 23 °C. Mice were allowed free access to regular food and water. Animal experiments were approved by the animal ethics committee of the Jiangsu University. Study methods were carried out in accordance with relevant guidelines and regulations.

### Mouse model of Ang II-induced cardiac hypertrophy

The continuous infusion of Ang II was achieved by using an indwelling, chronically implanted osmotic mini-pump (model 2004, ALZET Technical Services, USA) as previously described^36^. Specifically, the mini-pump was subcutaneously embedded in mice under anesthesia condition (80 mg/kg ketamine and 8 mg/kg xylazine). Ang II (1 μg/kg/min) or normal saline was infused constantly for four weeks. 3 days before implantation of osmotic mini-pump, mice were intraperitoneally injected with AS-1 (50 mg/kg/day) for 4 weeks. AS-1 was prepared by mixing one volume of AS-1 in DMSO with three volumes of saline to give a final concentration of 50 mg/kg body weight. Vehicle control was prepared by mixing one volume of DMSO with three volumes of saline. Four weeks after Ang II infusion, hearts were harvested and the ratio of heart weight/body weight (HW/BW) and left ventricular weight/tibia length (LVW/TL) were calculated. The harvested heart samples were processed for further analysis. Experimental mice were assigned to four groups according to different treatments: sham control (sham), Ang II-induced (Ang II), Ang II-induced + DMSO, Ang II-induced + AS-1.

### Echocardiography and tail cuff blood pressure measurement

Cardiac functions were evaluated by echocardiography using a 40 MHz transducer and a VEVO 2100 console (VisualSonics, Toronto, ON, Canada). Echocardiography was obtained on anesthetized mice 4 weeks after implantation of osmotic mini-pump. M-mode tracings were used to measure intraventricular septum thickness in diastole (IVS, d) and thickness of the rear wall of the left ventricular (LVPW, d). Echocardiography was done by an experienced echocardiographer in a double-blind manner. All measurements were averaged over six consecutive cardiac cycles. Mouse blood pressure was determined by the measurement of tail cuff blood pressure and averaged over three measurements.

### Cell culture

Primary cultures of cardiomyocytes were isolated from 1 to 3-day-old neonatal rats as described previously^18^. Briefly, after sacrificing, the hearts of newborns were removed under sterile condition. Ventricular tissues were excised, minced and digested with 0.08% trypsin (Gibco, USA). Cells were centrifuged at 1,800 rpm/min for 5 min and resuspended in Dulbecco’s Modified Eagle’s Medium (DMEM) (Gibco, USA) supplemented with penicillin/streptomycin (Beyotime Biotechnology, China) and 10% fetal bovine serum (FBS) (Gibco, USA). Primary cells were induced with Ang II (0.1 μM) for 24 hours. AS-1 (100 μM) or DMSO was administrated 30 min before Ang II application.

### Synthesis and administration of antagomirR-143

The micrOFFTM mmu-miR-143-3p antagomirR (antagomiR-143) and scramble siRNA were purchased and synthesized by RiboBio (Guangzhou, China).

### Overexpression of miRNA-143

Control miRNA and miRNA-143 mimics were synthesized and obtained from Guangzhou RiboBio (Guangzhou, China). The miRNA was transfected into the primary cardiomyocytes using Lipofectamine 3000 (Invitrogen, USA) according to the manufacturer’s instructions. The sequences used in this study are as follows: miRNA control, 5′-UCACAACCUCCUAGAAAGAGUAGA-3′; rno-miR-143-3p mimics, 5′-UGAGAUGAAGCACUGUAGCUCAUGAGCUACAGUGCUUCAUCUCA-3′.

### Quantitative real-time PCR (qRT-PCR)

RNA levels were measured by qRT-PCR. Briefly, RNAs of primary cardiomyocytes were extracted using RNAiso reagent (Takara Biotechnology). Subsequently, total RNA was reverse transcribed to complementary DNA (cDNA) according to the protocol of PrimeScript^TM^ RT detection kit (for mRNA) or PrimeScript^TM^ miRNA RT detection kit (for miRNA) (Takara Biotechnology). The RT primer of miRNA-143: 5′ GTCGTATCCAGTGCGTGTCGTGGAGTCGGCAATTGCACTGGATACGACTGAGCT 3′; RT primer of U6: 5′ CGCTTCACGAATTTGCGTGTCAT 3′. qPCR Primer sets are indicated in Table 1. All primers were synthesized by Sangon Biotech (Shanghai, China). qPCR reactions were performed using the SYBR Premix Ex Taq^TM^ II kit as described by the protocol. The same thermocycling profile was used for all genes: 95 °C for 10 minutes, followed by 40 cycles of 95 °C for 15 seconds, 58 °C for 30 seconds, and 72 °C for 20 seconds.

**Table 1:** List of primers.

Gene	Forward 5′-3′	Rerverse 5′-3′
ANP	ATACAGTGCGGTGTCCAACA	CGAGAGCACCTCCATCTCTC
BNP	GCTTTGGGCAGAAGATAGA	CAAGTTTGTGCTGGAAGATAA
β-MHC	TGCTGGCACCGTGGACTA	GCTTGAGGGAGGACTTCTGG
GAPDH	GCCAGCCTCGTCTCATAGACA	AGAGAAGGCAGCCCTGGTAAC
U6	CTCGCTTCGGCAGCACA	AACGCTTCACGAATTTGCGT
mir143	GGGTGAGATGAAGCACTGT	CAGTGCGTGTCGTGGAGT

### Immunofluorescence staining

Cells were rinsed three times in pre-cold PBS, followed by fixation using 4% paraformaldehyde (PFA)/phosphate-buffered saline (PBS) for 20 minutes at room temperature. Cells were permeabilized by 0.1% Triton X-100 for 20 minutes followed by blocking with 3% bovine serum albumin (BSA) for 30 minutes at room temperature. Then, cells were incubated at 4 °C overnight with anti-α-actinin (Sigma) or p50 NF-κB (CST) primary antibody. The Alexa Fluor 488-conjugated secondary antibody (1:500) was used to visualize α-actinin. The Alexa Fluor 594-conjugated secondary antibody (1:500) was used to visualize p50. The images were taken under a fluorescence microscope (Olympus). Confocal images were acquired with a laser-scanning confocal microscope (Zeiss LSM 510 META).

### Wheat germ agglutinin (WAG) staining

The saline-perfused mouse hearts were fixed in 4% PFA and embedded for paraffin blocks. Paraffin sections were prepared according to published protocols. To visualize cellular borders, dewaxed paraffin sections were incubated with WAG working buffer (5 μg/ml) for 1 hour. After rinse with PBS for three times, sections were mounted with a drop of mounting medium. Images were taken under a fluorescence microscope (Olympus).

### Western blot

Primary cardiomyocytes were lysed with ice-cold RIPA lysis buffer. Western blot was performed as described elsewhere^17^. Briefly, proteins were separated by SDS-PAGE and transferred onto polyvinylidene difluoride (PVDF) membranes (Amersham Biosciences, USA). The PVDF membranes were incubated with anti-ERK1/2 and anti-phospho-ERK1/2 antibodies (Cell Signaling Technology, USA), followed by incubation with peroxidase-conjugated secondary antibody. The signals were detected with the ECL system (Pierce, USA). Qualification of signals were performed by ImageJ software.

### Statistics

All data are expressed as means ± SEM (standard error of the mean). Statistical analyses were performed by ANOVA and the Tukey posttest procedure, with P < 0.05 considered significant.

## Data Availability

The datasets generated during and/or analysed during the current study are available from the corresponding author on reasonable request.
